# Using unsupervised clustering approaches to identify common mental health profiles and associated mental health-care service-use patterns in Ontario, Canada

**DOI:** 10.1093/aje/kwae030

**Published:** 2024-04-03

**Authors:** Christa Orchard, Elizabeth Lin, Laura Rosella, Peter M Smith

**Keywords:** mental health, health-care service use, machine learning, unsupervised learning, administrative data, survey data, psychiatry, psychology

## Abstract

Mental health is a complex, multidimensional concept that goes beyond clinical diagnoses, including psychological distress, life stress, and well-being. In this study, we aimed to use unsupervised clustering approaches to identify multidimensional mental health profiles that exist in the population, and their associated service-use patterns. The data source was the 2012 Canadian Community Health Survey–Mental Health, linked to administrative health-care data; all Ontario, Canada, adult respondents were included. We used a partitioning around medoids clustering algorithm with Gower’s proximity to identify groups with distinct combinations of mental health indicators and described them according to their sociodemographic and service-use characteristics. We identified 4 groups with distinct mental health profiles, including 1 group that met the clinical threshold for a depressive diagnosis, with the remaining 3 groups expressing differences in positive mental health, life stress, and self-rated mental health. The 4 groups had different age, employment, and income profiles and exhibited differential access to mental health-care services. This study represents the first step in identifying complex profiles of mental health at the population level in Ontario. Further research is required to better understand the potential causes and consequences of belonging to each of the mental health profiles identified.

This article is part of a Special Collection on Mental Health.

In Ontario, Canada, mental health care provided in general and specialist hospitals, as well as by physicians, including psychiatrists, is covered for all residents through the Ontario Health Insurance Plan (OHIP). Other types of mental health care, including psychological services and community-based care, are paid for privately, paid for out of pocket, or funded publicly outside of OHIP. In 2012, 1.6 million adults accessed publicly funded mental health outpatient care in Ontario, 130 000 accessed emergency care, and 50 000 accessed inpatient care.^1^ In order to understand who may access and benefit from publicly funded mental health care, clinical experts have created mental health diagnoses representing combinations of symptoms with defined thresholds of severity, duration, and impact on functioning.^2^ Using these diagnostic definitions, approximately 7 million Canadians and just under 1 in 10 Ontarians live with a mental or substance-use disorder.^3^^,^^4^

However, mental health is a complex, multidimensional concept that is greater than simply the absence of symptoms of mental illness.^5^^,^^6^ Diagnoses are often difficult to clearly identify, as there is a substantial degree of overlap in symptoms across disorders, along with high rates of comorbidity.^7^^-^^9^ Experiencing multiple mental health disorders has been linked to increased risk of suicide and unique care needs.^10^^-^^12^ Conversely, only half of mental health service users have a diagnosable mental illness, suggesting this is not the only dimension of mental health that is relevant to understanding why individuals access care.^13^^-^^15^ Experiencing a subthreshold disorder, psychological distress, low self-perceived mental health, and life stress are recognized dimensions of mental health that have been linked to increased service use independent of diagnoses.^13^^,^^14^^,^^16^^-^^20^

Positive mental health is also an important dimension of mental health that is orthogonal to mental illness; it represents the social, emotional, and psychological well-being of an individual.^5^^,^^6^^,^^21^ A person with a diagnosable mental disorder can experience the presence or absence of well-being, and some studies have shown that persons with a mental illness who are deemed to be “flourishing” in their positive mental health have better functioning and resilience, as well as better mental health outcomes, including less suicidal behavior.^22^^,^^23^

Because the manifestation of mental health within individuals is complex, it is not best captured using single diagnostic indicators. In addition, different combinations of dimensions of mental health or “mental health profiles” could be linked to different patterns of use of mental health-care services. Understanding mental health profiles among the population and how they are linked to mental health service-use patterns could therefore be highly useful to informing mental health-care planning.

Population segmentation is an approach that enables the grouping of individuals within a population who have similar characteristics, such as health-care patterns or health symptoms.^24^ While initially segmentation was performed on the basis of clinical experience, data-driven methods, such as novel unsupervised clustering methods, can parse through complex data to find patterns and relationships that are not limited to a priori groups defined by clinicians.^25^^,^^26^ The goal of data-driven segmentation is to identify groups with unique service-use needs or service use, for whom care can be planned, and to inform policies and interventions designed to improve care experiences.^26^^-^^28^

To date, population segmentation has primarily been used to characterize general health-care service use and needs; however, an increasing number of studies have used segmentation approaches specific to mental health.^29^^-^^32^ No population segmentation tools for mental health exist yet in Ontario. In addition, most existing tools in other contexts focus on clinical populations, missing those who may have an unmet need for care. Tools also tend to focus on diagnoses rather than capture all aspects of mental health, and very few adequately capture service use beyond cross-sectional or single measures.^33^^-^^37^ Further, segmentation tools often incorporate clinical needs and service use into a single tool. Yet, in the context of mental health care, needs are complex, are difficult to define, and are not always well aligned with service use.^20^^,^^32^^,^^34^^,^^38^^-^^40^ Although the factors contributing to service-use needs extend beyond clinically defined mental health to social determinants of health, services are funded and mandated to treat mental health problems. Through segmenting mental health profiles separately from care patterns, we can identify how service use tends to unfold for groups with similar profiles.

Establishing mental health profiles within the Ontario population along with their associated service-use patterns can inform the development of care pathways and programs designed to meet the service needs of these groups. These segments can also be used to inform policies and strategies that are targeted towards specific mental health profiles.

Therefore, the objective of this research was to use unsupervised clustering approaches to identify and characterize groups with unique mental health profiles in the Ontario population, and to identify the mental health service-use patterns associated with these profiles.

## Methods

### Data source and population

The primary data source for this study was the 2012 Canadian Community Health Survey, Mental Health edition (CCHS-MH), a cross-sectional survey disseminated to a representative sample of Canadians aged ≥15 years living in private dwellings.^41^ To characterize mental health-care service use in the eligible cohort, the CCHS-MH data were linked with administrative data holdings available through Data & Analytic Services at the Institute for Clinical Evaluative Sciences (ICES), including the Registered Persons Database, the Ontario Mental Health Reporting System, the Canadian Institute for Health Information’s (CIHI) Discharge Abstract Database, the National Ambulatory Care Reporting System, and OHIP.

All OHIP-eligible and enrolled adults aged ≥16 years who completed the 2012 CCHS-MH survey were included. Those under age 16 were excluded, since they were likely to access specialized child and adolescent mental health services funded outside of OHIP. Given that the age range of the CCHS-MH participants was 15 years or older, this excluded only a small number of individuals. Those who were ineligible for OHIP within 12 months of their CCHS-MH interview date in 2012 were excluded using the “OHIPELIG” macro developed by ICES.

### Measurement

#### Input variables

The variables included in the clustering algorithm are described in Table 1. All measures were captured in a single interview conducted between January and December 2012. Input variables were selected from the CCHS-MH measures to capture different dimensions or aspects of mental health that have previously been linked to likelihood of accessing services. This included clinically diagnosable mental health conditions, indicators of stress, and negative mental health symptoms that may not meet the criteria for a mental health disorder (psychological distress, life stress), indicators of well-being (life satisfaction, self-rated mental health), and a measure of positive mental health (the Mental Health Continuum Short Form).^6^^,^^18^ Where available, validated measures were selected; in their absence, single-item measures were selected. To ensure statistically that the different measures were capturing different constructs, we examined the correlation between measures to ensure lack of complete overlap.

**Table 1:** Variables in the 2012 Canadian Community Health Survey–Mental Health selected as clustering input variables.

**Concept**	**Instrument (if applicable)**	**Description**
Mood disorder	WMH-CIDI instrument^42^	Met DSM-IV^2^ criteria for past-year depression and/or bipolar disorder
Anxiety disorder	WMH-CIDI instrument	Met DSM-IV criteria for past-year generalized anxiety disorder
Substance-use disorder	WMH-CIDI instrument	Met DSM-IV criteria for past-year alcohol, cannabis, or other substance abuse and dependence
Schizophrenia or psychosis		Single-item self-reported current diagnosis
Eating disorder		Single-item self-reported current diagnosis
Suicidal thoughts or attempts in past 12 months		Single items indicating serious thoughts, attempts, or plans for suicide in the past 2 weeks or the past 12 months
Psychological distress	6-item Kessler Psychological Distress Scale^18^	Score range, 0-24; higher scores indicate greater psychological distress, which is often an indicator of mental illness
Self-rated mental health		Single-item general self-assessment of mental health
Life stress		Single-item amount of stress in life on most days
Positive mental health	Mental Health Continuum Short Form^6^	Continuous score used to classify individuals into one of 3 groups

Abbreviations: DSM-IV, *Diagnostic and Statistical Manual of Mental Disorders, Fourth Edition*; WMH-CIDI, World Mental Health Composite International Diagnostic Interview.

#### Other CCHS-MH covariates

Sociodemographic variables such as age, ethnicity, educational attainment, immigrant status, and income are self-reported within the CCHS-MH. Geographic region (urban/rural) was measured using Statistics Canada’s definition based on population density and census metropolitan area.^43^

#### Mental health service use

Episodes of mental health or addiction-related care received by the eligible participants between January 1 and December 31, 2012, were identified using the administrative data available at ICES. All episodes of care within the Ontario Mental Health Reporting System were captured, while primary diagnostic codes were used to identify mental health or addiction-related inpatient visits within the CIHI Discharge Abstract Database and emergency care visits in the National Ambulatory Care Reporting System (diagnostic codes available in Tables S1-S3), and an existing algorithm was used to identify outpatient visits in OHIP data, with 80.7% specificity and 97.0% sensitivity.^44^ Care received was subsequently summarized for each individual as the total count of (1) family physician visits, (2) psychiatrist visits, (3) emergency care visits, (4) hospitalizations, and (5) other specialist visits.

### Analyses

In order to identify clusters with similar mental health profiles, a partitioning around medoids (PAM) algorithm was used.^45^ An extension of the *k*-means algorithm, the PAM procedure is a centroid-based clustering algorithm that uses an iterative process to identify clusters; each individual is first randomly assigned to a cluster, the cluster center is then calculated, and the proximity between each individual and each cluster center is measured, with individuals being assigned to the nearest cluster. This process is repeated until no changes in cluster membership occur. As opposed to the *k*-means algorithm, which uses the mean as the cluster center, the PAM algorithm uses medoids to define the cluster center, which is robust to extreme outliers.^45^ In the context of this study, since there may have been a small group of individuals with very poor mental health in the sample, this approach ensured that they did not have undue influence on the groupings identified, allowing us to identify variation between the majority of individuals. The Gower’s proximity function was used to define proximity between each individual and the cluster center, because it adapts to different types of data, including continuous, ordinal, and nominal/binary data, to produce an overall measure of distance between 0 (identical) and 1 (maximally dissimilar).^46^ This clustering approach has been used previously to find groups using health-related administrative and survey data.^47^^,^^48^

Since the PAM algorithm requires a prespecified number of clusters, it was repeated with 2-8 clusters, and internal cluster validity was assessed using the silhouette coefficient, a measure that captures the average similarity between individuals and their own cluster as compared with other clusters.^49^ In addition to quantitative assessment of cluster performance, the utility and meaningfulness of the clustering solution was assessed, and both were weighed in selecting a final solution. Once the final clustering solution was identified, the clusters were characterized according to the input variables and assigned a summary label indicating the mental health profile they were deemed to represent. The final clustering solution was characterized using the sociodemographic variables available in the CCHS-MH.

Among individuals with any mental health or addiction-related care episode identified within the ICES administrative data, a similar clustering approach was used to identify service-use patterns. Specifically, a PAM algorithm was used to identify clusters with similar mental health service-use patterns. However, for services, a Bray-Curtis distance was used to accommodate the count data (numbers of each type of service use).^45^^,^^50^ The solution that represented the best balance between cluster validity (measured using the silhouette coefficient) and utility was selected, using a similar approach as that used to identify mental health profile clusters. These clusters were used to characterize mental health service-use patterns among the different mental health profiles identified within the CCHS-MH data. An alluvial plot was produced to visualize the relationship between mental health profiles identified within the CCHS-MH and service-use patterns identified within the ICES administrative data.

Since the PAM algorithm is subject to random variation depending on the initial seed used to select cluster centers, both clustering algorithms were repeated with different seeds, as well as with trimmed values of extreme outliers (each count was trimmed at the 99.9th percentile). An adjusted Rand index was used to compare solutions, a measure of agreement across pairs of individuals adjusted for agreement by chance.^33^^,^^34^ A high degree of similarity across solutions (most individuals are in the same cluster in both solutions) indicates that the cluster solution is robust against analytical decisions.

## Results

In total, 4159 Ontario residents were identified among the 2012 CCHS-MH survey participants, of whom 73 (1.8%) were excluded due to being under 16 years of age at the time of interview, and 85 (2.0%) were excluded due to lacking OHIP eligibility within 12 months of their CCHS interview date. An additional 191 (4.6%) individuals were missing data on 1 or more input variables and were therefore excluded, leaving a final analytical sample of 3810. Sociodemographic and mental health characteristics of the analytical sample are shown in Table 2. Overall, the average age of the sample was 49 years, with a relatively even spread across age groups; 53% were female, and 83% resided in urban areas. Educational levels were high, with over two-thirds of participants having a postsecondary education, and more than half were employed in some capacity.

**Table 2:** Sociodemographic and mental health characteristics of a cohort of Ontario, Canada, adults (*n* = 3810), Canadian Community Health Survey–Mental Health, 2012.

**Variable**	**No. (%)** ^**a**^
Age, y	
Mean (SD)	48.5 (19.7)
Median (range)	49.0 (16.0-99.0)
Age group, y	
15-24	600 (15.7)
25-34	487 (12.8)
35-44	568 (14.9)
45-54	577 (15.1)
55-64	659 (17.3)
≥65	919 (24.1)
Sex	
Female	2028 (53.2)
Male	1782 (46.8)
Geographic region	
Population center	3143 (82.5)
Rural area	667 (17.5)
Highest level of education	
Less than secondary school graduation	350 (9.2)
Secondary school graduation	470 (12.3)
Some postsecondary school	144 (3.8)
Postsecondary school graduation	2637 (69.2)
Marital status	
No (not married)	1718 (45.1)
Yes (married and/or common law)	2086 (54.8)
Recent immigrant (past 10 years)	
No	3643 (95.6)
Yes	162 (4.3)
Employment	
Full-time	1776 (46.6)
Part-time	426 (11.2)
Presumed unemployed	1603 (42.1)
Income, CAD$/y	
<40 000	1189 (31.2)
40 000-59 999	710 (18.6)
60 000-79 999	567 (14.9)
80 000-99 999	420 (11.0)
100 000-149 999	558 (14.6)
≥150 000	366 (9.6)
Ethnicity	
White	3056 (80.2)
Black	118 (3.1)
East or Southeast Asian	224 (5.9)
South Asian	157 (4.1)
Latino	61 (1.6)
Middle Eastern	55 (1.4)
Mental health diagnosis	
Mood disorder	230 (6.0)
Anxiety disorder	110 (2.9)
Eating disorder	20 (0.5)
Schizophrenia	49 (1.3)
Substance-use disorder	168 (4.4)
Suicidal thoughts or attempts	149 (3.9)
Self-rated mental health	
Excellent	931 (24.4)
Very good	1514 (39.7)
Good	1022 (26.8)
Fair	275 (7.2)
Poor	68 (1.8)
Level of life stress	
Not at all stressful	523 (13.7)
Not very stressful	942 (24.7)
A bit stressful	1578 (41.4)
Quite a bit stressful	657 (17.2)
Extremely stressful	110 (2.9)
Psychological distress score^b^	
Mean (SD)	3.1 (3.5)
Median (range)	2.0 (0.0-24.0)
Positive mental health^c^	
Languishing	76 (2.0)
Moderate	829 (21.8)
Flourishing	2905 (76.2)

^a^Data presented are numbers and percentages unless otherwise indicated.

^b^0 = lowest, 24 = highest.

^c^Mental Health Continuum Short Form positive mental health scale.

Generally mental health was positive in this sample, with the majority (over 90%) describing their mental health as good, very good, or excellent, and over three-quarters were “flourishing” according to the Mental Health Continuum Short Form positive mental health scale. Clinically diagnosable mental health disorders were rare, the most common being a mood disorder, which occurred among 6% of the sample. Most of the sample expressed at least some life stress, and around 1 in 5 participants in the sample felt they were under quite a bit of stress or extreme stress.

**Figure 1:**
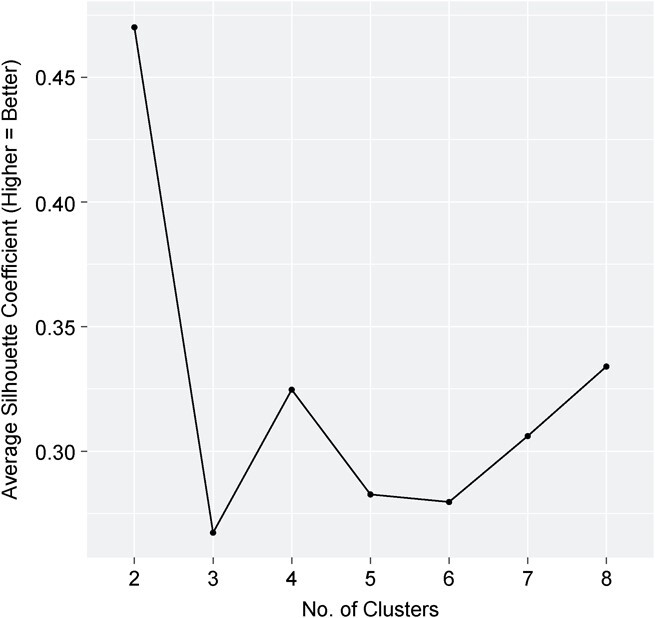
Silhouette plot displaying internal cluster validity by *k*-cluster for a mental health clustering solution using data from the Ontario component of the Canadian Community Health Survey–Mental Health, 2012.

**Figure 2:**
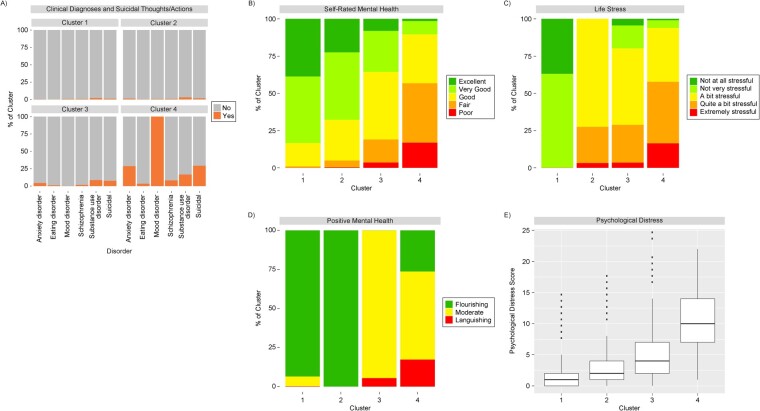
Visualization of a 4-cluster solution identifying mental health profiles according to mental-health–related input variables, Ontario component of the Canadian Community Health Survey–Mental Health, 2012. A) Clinical diagnoses and suicidal thoughts/actions; B) self-rated mental health; C) life stress; D) positive mental health; E) psychological distress (0 = lowest score, 24 = highest score). Cluster 1 (*n* = 1321)—flourishing, minimal/no life stress; cluster 2 (*n* = 1615)—flourishing, some life stress; cluster 3 (*n* = 663)—moderate mental health and stress; cluster 4 (*n* = 211)—clinical mood disorder. The box-and-whisker plot in panel E was constructed using the Tukey method.^51^

Figure 1 displays a silhouette plot showing the silhouette coefficients for the PAM algorithm by *k*-cluster. The 2-cluster solution produced the highest internal validity, and there was a second peak at 4 clusters, followed by a third peak at 8 clusters. After examining the clustering solutions according to their input variables, we deemed the 2-cluster solution to be of limited utility, primarily representing the presence or absence of positive mental health. The 8-cluster solution produced a finer-grained solution that began to identify all possible combinations of the input variables, but it was lacking in parsimony/utility due to the size of the solution. Therefore, the 4-cluster solution was deemed to represent the best balance between internal validity and usefulness, given its simplicity and ability to capture distinguishable groups. A visualization of the input variables across the 4 clusters is shown in Figure 2, and the 2-cluster and 8-cluster solutions are depicted in Figures S1 and S2, respectively. Sociodemographic information and input variables are displayed across the clusters in Table 3.

**Table 3:** Distribution (no. (%)^a^) of participants into clusters with distinct mental health profiles as determined by sociodemographic and mental health characteristics (*n* = 3810), Canadian Community Health Survey–Mental Health, 2012.

**Variable**	**Cluster**			
	**Cluster 1 (flourishing, minimal/no life stress; *n* = 1321)**	**Cluster 2 (flourishing, some life stress; *n* = 1615)**	**Cluster 3 (moderate mental health and stress; *n* = 663)**	**Cluster 4 (clinical mood disorder; *n* = 211)**
Age, y				
Mean (SD)	54.1 (20.8)	45.7 (18.1)	46.4 (19.3)	42.4 (16.5)
Median (range)	59.0 (16.0-99.0)	46.0 (16.0-93.0)	45.0 (16.0-97.0)	44.0 (16.0-88.0)
Age group, y				
15-24	183 (13.9)	263 (16.3)	114 (17.2)	40 (19.0)
25-34	118 (8.9)	231 (14.3)	103 (15.5)	35 (16.6)
35-44	140 (10.6)	284 (17.6)	109 (16.4)	35 (16.6)
45-54	134 (10.1)	300 (18.6)	92 (13.9)	51 (24.2)
55-64	241 (18.2)	275 (17.0)	113 (17.0)	30 (14.2)
≥65	505 (38.2)	262 (16.2)	132 (19.9)	20 (9.5)
Sex				
Female	672 (50.9)	911 (56.4)	314 (47.4)	131 (62.1)
Male	649 (49.1)	704 (43.6)	349 (52.6)	80 (37.9)
Geographic region				
Population center	1063 (80.5)	1320 (81.7)	569 (85.8)	191 (90.5)
Rural area	258 (19.5)	295 (18.3)	94 (14.2)	20 (9.5)
Highest level of education				
Less than secondary school graduation	158 (12.0)	110 (6.8)	61 (9.2)	21 (10.0)
Secondary school graduation	189 (14.3)	169 (10.5)	90 (13.6)	22 (10.4)
Some postsecondary school	40 (3.0)	54 (3.3)	31 (4.7)	19 (9.0)
Postsecondary school graduation	860 (65.1)	1191 (73.7)	447 (67.4)	139 (65.9)
Marital status				
No (not married)	607 (46.0)	662 (41.0)	334 (50.4)	115 (54.5)
Yes (married)	712 (53.9)	952 (58.9)	328 (49.5)	94 (44.5)
Recent immigrant (past 10 years)				
No	1262 (95.5)	1538 (95.2)	637 (96.1)	206-210^b^ (97.6-99.5)
Yes	57 (4.3)	75 (4.6)	25 (3.8)	1-5^b^ (0.5-2.4)
Employment				
Full-time	473 (35.8)	928 (57.5)	294 (44.3)	81 (38.4)
Part-time	150 (11.4)	177 (11.0)	71 (10.7)	28 (13.3)
Presumed unemployed	696 (52.7)	507 (31.4)	298 (44.9)	102 (48.3)
Income, CAD$/y				
<40 000	426 (32.2)	419 (25.9)	239 (36.0)	105 (49.8)
40 000-59 999	263 (19.9)	276 (17.1)	142 (21.4)	29 (13.7)
60 000-79 999	192 (14.5)	264 (16.3)	85 (12.8)	26 (12.3)
80 000-99 999	140 (10.6)	193 (12.0)	68 (10.3)	19 (9.0)
100 000-149 999	183 (13.9)	272 (16.8)	80 (12.1)	23 (10.9)
≥150 000	117 (8.9)	191 (11.8)	49 (7.4)	9 (4.3)
White ethnicity	1066 (80.7)	1284 (79.5)	534 (80.5)	172 (81.5)
Mental health diagnosis				
Mood disorder	10 (0.8)	9 (0.6)	0 (0.0)	211 (100.0)
Anxiety disorder	1-5^b^ (0-0.4)	19 (1.2)	28 (4.2)	60 (28.4)
Substance-use disorder	28 (2.1)	49 (3.0)	56 (8.4)	35 (16.6)
Schizophrenia	8 (0.6)	14 (0.9)	10 (1.5)	17 (8.1)
Eating disorder	1-5^b^ (0-0.4)	1-5^b^ (0-0.3)	6 (0.9)	7 (3.3)
Suicidal thoughts or attempts	12 (0.9)	27 (1.7)	48 (7.2)	62 (29.4)
Psychological distress score^c^				
Mean (SD)	1.4 (1.8)	2.7 (2.6)	5.2 (4.0)	10.1 (4.6)
Median (range)	1.0 (0.0-13.0)	2.0 (0.0-16.0)	4.0 (0.0-24.0)	10.0 (1.0-22.0)
Self-rated mental health				
Excellent	511 (38.7)	366 (22.7)	51 (7.7)	1-5^b^ (0.5-2.4)
Very good	590 (44.7)	722 (44.7)	183 (27.6)	17-21^b^ (8.1-10.0)
Good	206 (15.6)	446 (27.6)	301 (45.4)	69 (32.7)
Fair	12 (0.9)	75 (4.6)	104 (15.7)	84 (39.8)
Poor	1-5^b^ (0-0.4)	6 (0.4)	24 (3.6)	36 (17.1)
Level of life stress				
Not at all stressful	492 (37.2)	0 (0.0)	29 (4.4)	1-5^b^ (0.5-2.4)
Not very stressful	829 (62.8)	0 (0.0)	102 (15.4)	8-12 (3.8-5.7)
A bit stressful	0 (0.0)	1164 (72.1)	338 (51.0)	76 (36.0)
Quite a bit stressful	0 (0.0)	399 (24.7)	171 (25.8)	87 (41.2)
Extremely stressful	0 (0.0)	52 (3.2)	23 (3.5)	35 (16.6)
Positive mental health^d^				
Languishing	1-5^b^ (0-0.4)	0 (0.0)	37 (5.6)	36 (17.1)
Moderate	82-86^b^ (6.2-6.5)	0 (0.0)	626 (94.4)	119 (56.4)
Flourishing	1234 (93.4)	1615 (100.0)	0 (0.0)	56 (26.5)

^a^ Data presented are numbers and percentages unless otherwise indicated.

^b^ A range of numbers (and percentages) is given when the true number was 5 or less, to reduce reidentification risk for individuals (plus 1 additional row within measures to avoid recalculation).

^c^ 0 = lowest, 24 = highest.

^d^ Mental Health Continuum Short Form positive mental health scale.

### Clusters

#### Cluster 1 (n = 1321, 34.7%)—flourishing, minimal/no life stress

The first cluster, capturing just over one-third of the sample, was a group of participants who were mostly flourishing and absent of clinical mental health diagnoses, with very low psychological distress scores. Most of this group reported their mental health as very good or excellent, and almost all people in this group reported that their lives were not very stressful or not at all stressful. Compared with the other clusters, individuals in this group were slightly older, with over half aged 55 years or more, and approximately 1 in 5 resided in rural areas, the highest among the clusters. Over half were not currently employed and were possibly retired, given the older age profile of this group.

#### Cluster 2 (n = 1615, 42.4%)—flourishing, some life stress

The second cluster, which included the largest percentage of respondents of all the clusters, represented those who were flourishing in their mental health and largely absent of clinical mental diagnoses. Average self-rated mental health was slightly lower in this group than in cluster 1, although the majority still reported their mental health to be very good or excellent. This group differed from cluster 1 in terms of their life stress, with all reporting at least “a bit” of life stress and one-quarter rating their level of life stress as “quite a bit” or “extreme.” Cluster 2 had a high proportion of married individuals (59%), and this group had the highest proportion of employed individuals of all the clusters (68%). This group had a higher proportion of persons aged 35-55 years, which may explain their higher employment rates. However, this group also had the highest socioeconomic profile, with almost three-quarters reporting postsecondary school graduation, and over a quarter of this group reported an annual household income of CAD$100 000 or above.

#### Cluster 3 (n = 663, 17.4%)—moderate mental health and stress

Cluster 3 was a small cluster that represented a group with middling-to-poor mental health but largely without a clinically diagnosable mental illness. This group had an absence of positive mental health, with the majority receiving a rating of “moderate” and some “languishing.” A small proportion met the criteria for an anxiety disorder (4.2%) or a substance-use disorder (8.4%); however, for the most part this group was absent of clinical mental health diagnoses. Self-rated mental health was lower in this group than in the first 2 clusters, with most reporting that their mental health was “good” or “fair.” Most of this group reported at least some life stress, and they had higher average psychological distress scores compared with the first 2 clusters, though the scores were still relatively low. Cluster 3 was the only cluster that was majority male, with 53% identifying as such, and an even representation across age groups. This group also had a lower average household income, with 57% reporting earning CAD$60 000/y or less, despite the fact that this group had a higher proportion of individuals working full-time than cluster 1.

#### Cluster 4 (n = 211, 5.5%)—clinical mood disorder

Cluster 4 represented a small group of individuals who met the criteria for a clinically diagnosable mood disorder. Comorbid clinical conditions were common in this group, with over one-quarter meeting the criteria for an anxiety disorder and 1 in 6 meeting the criteria for a substance-use disorder. Almost a third of this group also reported suicidal thoughts. Self-rated mental health was lowest in this group, with most participants reporting their mental health as “fair” or “poor.” Almost all individuals in this group reported some life stress, and psychological distress scores were elevated in comparison with the other groups. While most participants in this group did not have positive mental health, around 1 in 4 were still “flourishing.” Cluster 4 had the youngest age profile of all the clusters, with an average age of 42 years and over a third under age 35 years. This group also had a higher proportion of female-identifying individuals than the other groups and were more likely to reside in urban areas. This group included a slightly lower proportion of recent immigrants and those who were married. Almost half of this group reported a household income of less than CAD$40 000/y.

### Mental health service-use patterns

**Figure 3:**
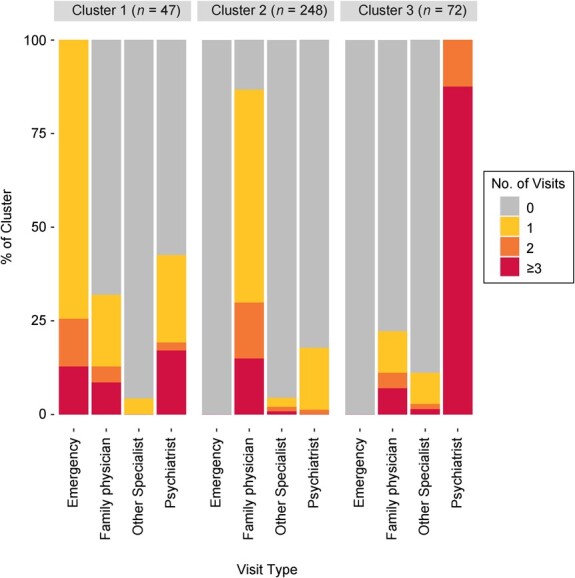
Visualization of a 3-cluster solution identifying mental health service-use patterns among respondents according to input variables, Canadian Community Health Survey–Mental Health, 2012.

In total, 367 (9.6%) of the 3810 eligible CCHS-MH respondents accessed publicly funded mental health services in the calendar year 2012. When the PAM clustering procedure was conducted in this group, the *k*-cluster with the highest silhouette coefficient was an 8-cluster solution. However, a 3-cluster solution was deemed to identify a meaningful and parsimonious summary of the service-use information with minimal loss of internal validity as compared with higher *k*-cluster solutions and was therefore selected (silhouette plot shown in Figure S3). The 3-cluster solution is presented in Figure 3. Cluster 1 (*n* = 47, 12.8% of service users), the smallest group, included participants with at least 1 emergency department visit in 2012, often paired with a visit to a family physician or psychiatrist. Cluster 2 (*n* = 248, 67.6%) represented two-thirds of all service users, and included those with minimal outpatient care, most often a family physician visit. Cluster 3 (*n* = 72, 19.6%) represented persons with multiple psychiatrist visits throughout the year, sometimes accompanied by other general outpatient care. Note that while persons who are hospitalized may represent an important group of mental health service users, hospitalizations were rare among CCHS-MH respondents, and therefore this variable did not have a substantial impact on the clustering solution and was excluded from the plots due to low numbers.

**Figure 4:**
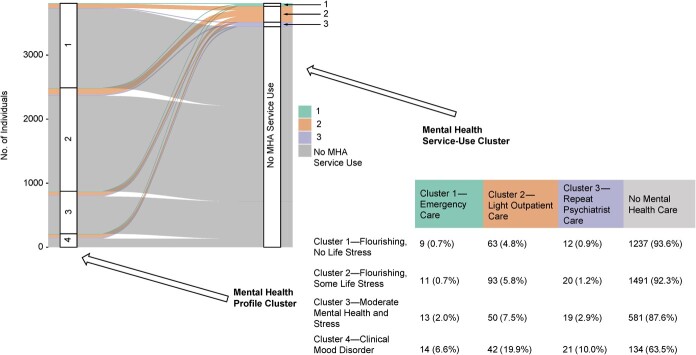
Alluvial plot and table demonstrating mental health service-use patterns across different mental health profiles of respondents, Canadian Community Health Survey–Mental Health, 2012. MHA, mental health and addictions.

Mental health service-use patterns, as represented by the 3 clusters identified, are presented by mental health profile in Figure 4. While most respondents in all groups did not access any mental health services, this varied across the 4 mental health profile clusters, and among service users, there appeared to be a gradient in access to increasingly intensive use of care with increasing negative mental health or stress. Despite both groups’ representing in large part those without a clinically diagnosable mental health disorder, those with moderate mental health and stress (cluster 3) were 3 times more likely to access repeated psychiatrist care, twice as likely to access emergency care, and 1.5 times as likely to access light outpatient care than those who were flourishing with no life stress (cluster 1). Participants with a clinical mood disorder had an elevated likelihood of accessing all types of care in comparison with the other 3 groups; however, almost two-thirds of this group did not access any type of mental health care, despite having elevated depressive symptoms that met the clinical threshold. These differences were tempered by small numbers.

## Discussion

This study demonstrates how an unsupervised clustering approach can identify more nuanced and complex groupings than single indicators alone, such as clinical diagnostic tools. We identified 4 mental health profiles among a representative sample of the general population in Ontario. One group was primarily defined by exhibiting clinically diagnosable depressive symptoms, while 3 of the 4 groups were mostly absent diagnosable mental health conditions but showed notable differences on mental health and well-being indicators that were linked to differential likelihood of service use. These mental health profiles were also found to have sociodemographic differences, including different age, employment, and income profiles.

Existing work that has been undertaken to examine mental health service use has typically focused on populations with a clinically diagnosable mental health disorder^28^^-^^31^ or the most intense and costly use.^52^ This study complemented and expanded on this work by focusing on a general population to better understand mental health and its relationship with mental health-care patterns. For example, we found that persons with moderate mental health but no clinically diagnosable disorder were more likely to access services than those who were flourishing and without life stress. We also found that approximately 1 in 20 of those who reported very good mental health received outpatient care, typically through a family doctor, while conversely over half of those with a diagnosable disorder did not access any care.

The reasons for these findings are complex; however, they do imply that current diagnostic indicators are only part of the picture in understanding mental health service use. While clinicians may not deem some of the mental health profiles we identified to represent persons who could benefit from treatment, it is clear that such individuals make up a nontrivial number of those accessing services. This may reflect a disconnect between clinician-assessed need (diagnostic indicators) and self-assessed need (indicated by access to services).^33^^,^^34^ It also builds upon existing work indicating that persons with symptoms that do not meet diagnostic thresholds exhibit substantial impairment over time and comprise a significant portion of those seeking primary care.^53^^-^^55^ In future work, researchers might attempt to understand self-assessed service-use needs among persons with the same mental health profile in order to determine the extent to which these needs are being met.

Beyond this, future work may seek to extrapolate and refine these profiles at the population level, by connecting these data with other information, such as long-term health care and economic data. Future work may also include qualitative interviews and consultations with clinical experts to identify relevant measurable outcomes specific to the population mental health profiles we identified, and to inform strategies and programs designed to improve these outcomes over time.

This study had some limitations. While the CCHS-MH identifies a representative sample of the general population, it is unlikely to capture individuals with severe or intense mental health problems or clinical needs. In addition, we identified publicly funded mental health services; however, due to lack of data, we could not capture or examine mental health services that are provided privately or through community-based programs. Therefore, it is not clear how these groups are accessing care outside of the public system. Finally, these were cross-sectional data; therefore, we could not assess whether participants accessing services had fewer symptoms due to receiving effective care. This may be more true for some patterns of service use (eg, repeated psychiatrist care) than for others (eg, emergency care or sparse general outpatient care). Future work with longitudinal data sets could also seek to expand our findings by examining the stability of profile membership over time.

In summary, this study represents the first step in modeling mental health as a multidimensional concept for identifying complex mental health profiles at the population level in Ontario. Further work is required to dig deeper into these profiles to inform strategies designed to improve outcomes within these groups.

## Supplementary Material

Web_Material_kwae030

## Data Availability

The data set from this study is held securely in coded form at ICES. While legal data-sharing agreements between ICES and data providers (eg, health-care organizations and government) prohibit ICES from making the data set publicly available, access may be granted to those who meet prespecified criteria for confidential access (website: www.ices.on.ca/DAS; email: das@ices.on.ca). The full data set creation plan and underlying analytical code are available from the authors upon request, understanding that the computer programs may rely upon coding templates or macros that are unique to ICES and therefore either are inaccessible or may require modification.

## Disclaimer

The opinions, results, and conclusions reported in this paper are those of the authors and are independent of the funding sources. No endorsement by ICES or the Ontario Ministry of Health is intended or should be inferred. This document uses data adapted from the Statistics Canada Postal Code Conversion File, which is based on data licensed from Canada Post Corporation, and/or data adapted from the Ontario Ministry of Health Postal Code Conversion File, which contains data copied under license from Canada Post Corporation and Statistics Canada. Parts of this material are based on data and/or information compiled and provided by CIHI and the Ontario Ministry of Health. The analyses, conclusions, opinions, and statements expressed herein are solely those of the authors and do not reflect those of the funding or data sources; no endorsement is intended or should be inferred. This document also uses data adapted from Statistics Canada (Canadian Community Health Survey–Mental Health, 2012). This does not constitute an endorsement of this product by Statistics Canada.
