# Supplementary material for: Using unsupervised clustering approaches to identify common mental health profiles and associated mental health-care service-use patterns in Ontario, Canada
Source: Am J Epidemiol. 2024 Apr 3;193(7):976–86. doi: 10.1093/aje/kwae030 (PMC11228863; doi:10.1093/aje/kwae030)
Supplement: Web_Material_kwae030 [file web_material_kwae030.pdf]

SUPPLEMENTARY MATERIAL

Using unsupervised clustering approaches to identify common mental health profiles and associated mental health-care service-use patterns in Ontario, Canada

Christa Orchard[1,2,3], Elizabeth Lin[3,4,5], Laura Rosella[1,3,6,7], Peter M Smith[1,2]

[1]Dalla Lana School of Public Health, University of Toronto, Toronto, Ontario, Canada

[2]Institute for Work & Health, Toronto, Ontario, Canada

[3]Institute for Clinical Evaluative Sciences, Ontario, Canada

[4]Centre for Addiction and Mental Health, Toronto, Ontario, Canada

[5]Department of Psychiatry, University of Toronto, Toronto, Ontario, Canada

[6]Institute for Better Health, Trillium Health Partners, Mississauga, Ontario, Canada

[7]Temerty Faculty of Medicine, University of Toronto, Toronto, Ontario, Canada

Contents:

Tables S1-S3 — Diagnostic codes used to identify mental health and addictions-related care

Figure S1 — Visualization of 2-cluster solution identifying mental health profiles among CCHS-MH respondents by input variables

Figure S2 — Visualization of 8-cluster solution identifying mental health profiles among CCHS-MH respondents by input variables

Figure S3 — Silhouette plot for mental health care service use PAM clustering procedure

Table S1. Qualifying diagnostic codes for outpatient mental health and addictions (MHA) visits

Note: Adapted from concept dictionary on MHA health care with removal of codes, including those related to development or intellectual disabilities (removed codes shown as crossed through).

Category	DXCODE	Description
**All individuals (ages 16-105)**		
Psychotic disorders	295	Schizophrenia
	296	Manic-depressive psychoses, involutional melancholia
	297	Other paranoid states
	298	Other psychoses
Non-psychotic disorders	~~300~~	~~Anxiety neurosis, hysteria, neurasthenia, obsessive-compulsive neurosis, reactive depression~~
	301	Personality disorders
	302	Sexual deviations
	306	Psychosomatic illness
	309	Adjustment reaction
	311	Depressive disorder
Substance use disorders	303	Alcoholism
	304	Drug dependence
Social problems	897	Economic problems
	898	Marital difficulties
	899	Parent-child problems
	900	Problems with aged parents or in-laws
	901	Family disruption/divorce
	902	Education problems
	904	Social maladjustment
	905	Occupational problems
	906	Legal problems
	909	Other problems of social adjustment
**Adolescents and youth (ages 16-25)**		
	291	Alcohol psychosis, delirium tremens, Korsakov's psychosis
	292	Drug psychosis
	~~299~~	~~Childhood psychoses (eg autism)~~
	307	Habit spasms, tics, stuttering, tension headaches, anorexia nervosa, sleep disorders, enuresis
	313	Behaviour disorders of childhood and adolescence
	314	Hyperkinetic syndrome of childhood
	~~315~~	~~Specific delays in development (eg dyslexia, dyslalia, motor retardation)~~

Table S2. Qualifying diagnostic codes for MHA emergency department visits and hospitalizations
Note: adapted from concept dictionary on MHA health care with removal of codes relating to developmental or intellectual disabilities.

	**ICD-9-CM codes (OMHRS DSM-IV)**	**ICD-10-CA codes (DAD/NACRS)**
Prior to 2016/17 fiscal year (used for period between start of 2012 to start of fiscal year 2016/17)	AXIS1_DSM4CODE_DISCH1 = Any OMHRS diagnosis (includes missing; **excludes** 290.x, 294.x, 299.x, 317.x, 318.x, 319.x)<br><br>Exclude if AXIS1_DSM4CODE_DISCH1 missing and PROVDX_DSM4CODE_ADM1 = 2	Primary diagnostic code in: DX10CODE1 = F06-69, ~~F70-89,~~ F90-F99 or DX10CODE2-DX10CODE10 = X60-X84, Y10-Y19, Y28 when DX10CODE1 ne F06-69, F90-F99<br><br>For DX10CODE2-10 = X60-X84, Y10-Y19, Y28 when DX10CODE1 ne F06-69, ~~F70-89,~~ F90-F99 (deliberate self-harm) please specify DXTYPE = alldx or DXTYPE = 9
	**ICD-9-CM codes (OMHRS DSM-V)**	**ICD-10-CA codes (DAD/NACRS)**
After 2016/17 fiscal year (used for period between start of fiscal year 2016/17 and end of 2017)	DSM5CODE_DISCH1 = Any OMHRS (includes missing; **excludes** 290.x, 294.0x-, 299.x, 317.x, 318.x, 319.x)<br><br>Exclude if DSM5CODE_DISCH1 missing and PROVDX_DSM5CODE_ADM1 = 17	Primary diagnostic code in: DX10CODE1 = F06-69, ~~F70-89,~~ F90-F99 or DX10CODE2-DX10CODE10 = X60-X84, Y10-Y19, Y28 when DX10CODE1 ne F06-69, F90-F99<br><br>For DX10CODE2-10 = X60-X84, Y10-Y19, Y28 when DX10CODE1 ne F06-69, F90-F99 (deliberate self-harm) please specify DXTYPE = alldx or DXTYPE = 9

Table S3. Diagnostic codes for clinical categories for MHA outpatient, emergency department visits and hospitalizations prior to 2016/17 fiscal year *adapted from MHA guidelines for incorporating MHA into HSE using ICES admin sources*

	**ICD-9-CM codes (OMHRS DSM-IV)**	**ICD-10-CA codes (DAD/NACRS)**
**Substance-Related Disorders (SUB_DIS)**	AXIS1_DSM4CODE_DISCH1 = 291.x, 292.x, 303.x, 304.x, 305.x<br><br>PROVDX_DSM4CODE_ADM1 = 4	DX10CODE1 = F55, F10 to F19
**Schizophrenia (SCHIZ_DIS)**	AXIS1_DSM4CODE_DISCH1 = 295.x, 297.x, 298.x<br><br>PROVDX_DSM4CODE_ADM1 = 5	DX10CODE1 = F20 (excluding F20.4), F22, F23, F24, F25, F28, F29, F53.1
Mood disorders (MOOD_DIS)	AXIS1_DSM4CODE_DISCH1 = 296.x<br><br>PROVDX_DSM4CODE_ADM1 = 6	DX10CODE1 = F30, F31, F32, F33, F34, F38, F39, F53.0
Anxiety disorders (ANX_DIS)	AXIS1_DSM4CODE_DISCH1 = 300.x, 308.x, 309.x<br><br>PROVDX_DSM4CODE_ADM1 = 7, 15	DX10CODE1 = F40, F41, F42, F43, F48.8, F48.9; F93.1, F93.2
**Other selected disorders (OTHER_MHA)**	AXIS1_DSM4CODE_DISCH1 = 301.x, 302.x, 307.x, 312.x, 313.x, 314.x, 787.x<br><br>PROVDX_DSM4CODE_ADM1 = 1, 12, 16	DX10CODE1 = F50.0, F50.1, F50.2, F50.3, F50.8, F50.9, F60, F61, F62, F68, F69, F21, F90, F91, F92, F93 (excluding F93.1, F93.2), F94, F95, F98
**Deliberate self-harm†** (SELF_HARM)	N/A (DAD/NACRS)	DX10CODE2-10 = X60-X84, Y10-Y19, Y28 when DX10CODE1 ne F06-F99
**Social problems (SOC_PROB)**	897.x, 898.x, 899.x, 900.x, 901.x, 902.x, 904.x, 905.x, 906.x, 909.x	NA

† Deliberate self-harm (or intentional self injury) is an external injury; please specify DXTYPE = alldx or DXTYPE = 9

Figure S1. Visualization of 2-cluster solution identifying mental health profiles among CCHS-MH respondents by input variables

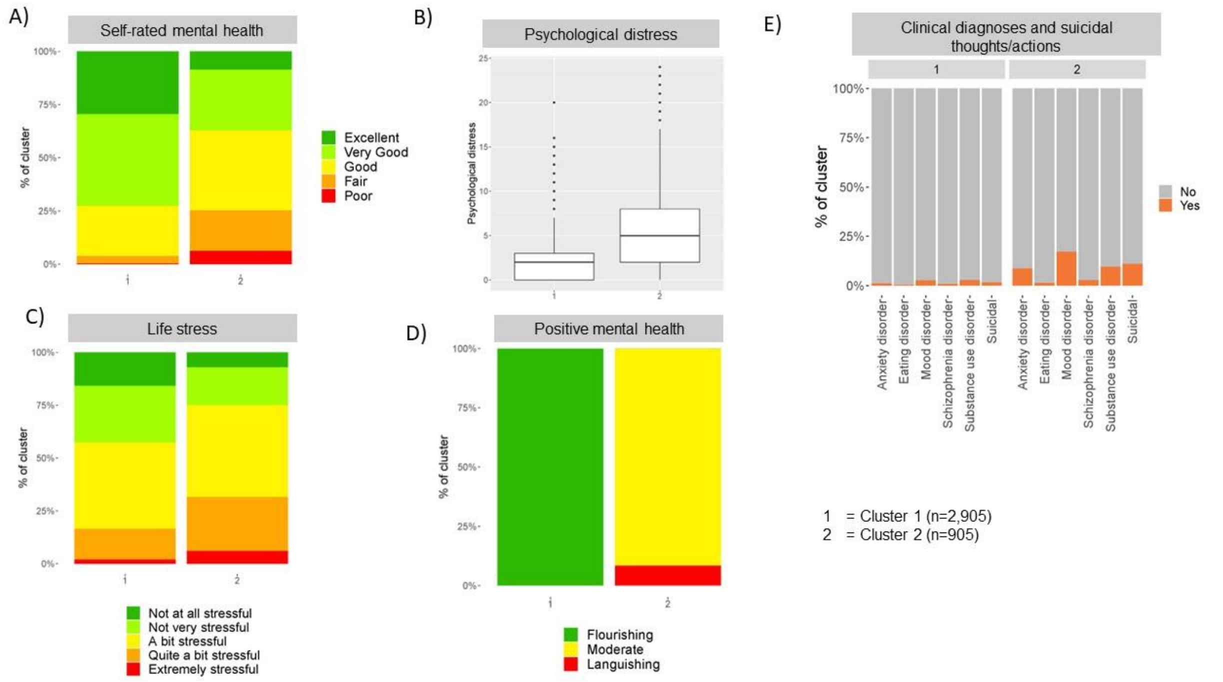

Figure S2. Visualization of 8-cluster solution identifying mental health profiles among CCHS-MH respondents by input variables

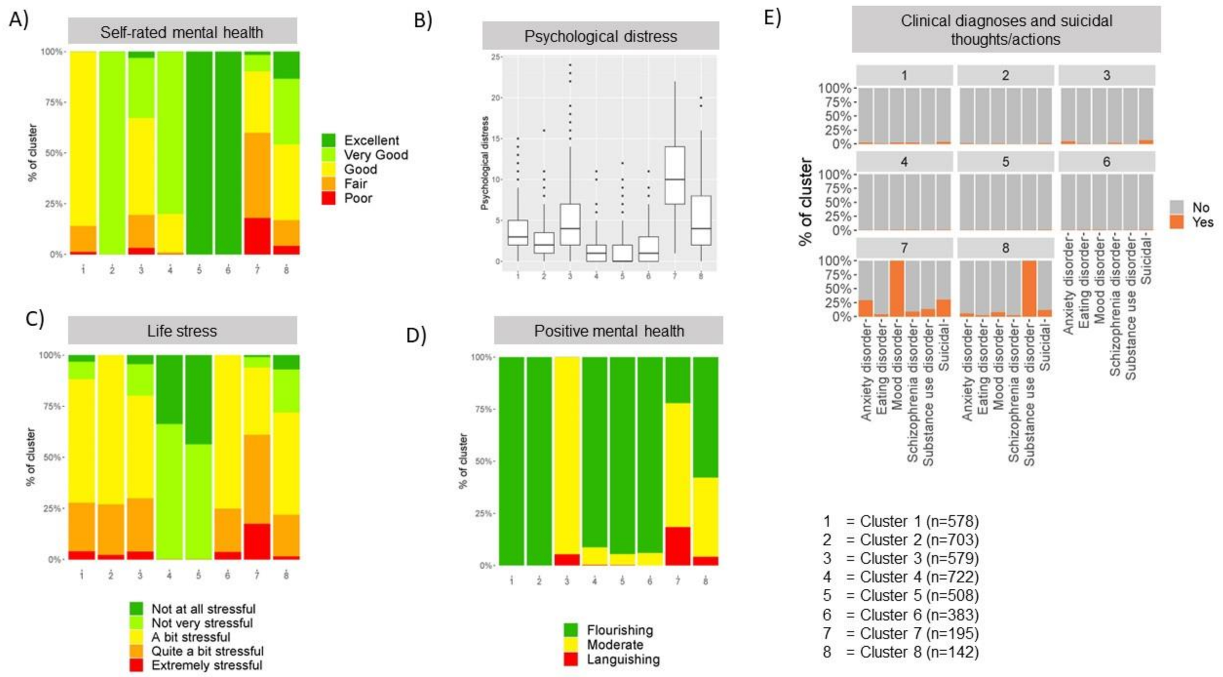

Figure S3. Silhouette plot for mental health care service use PAM clustering procedure

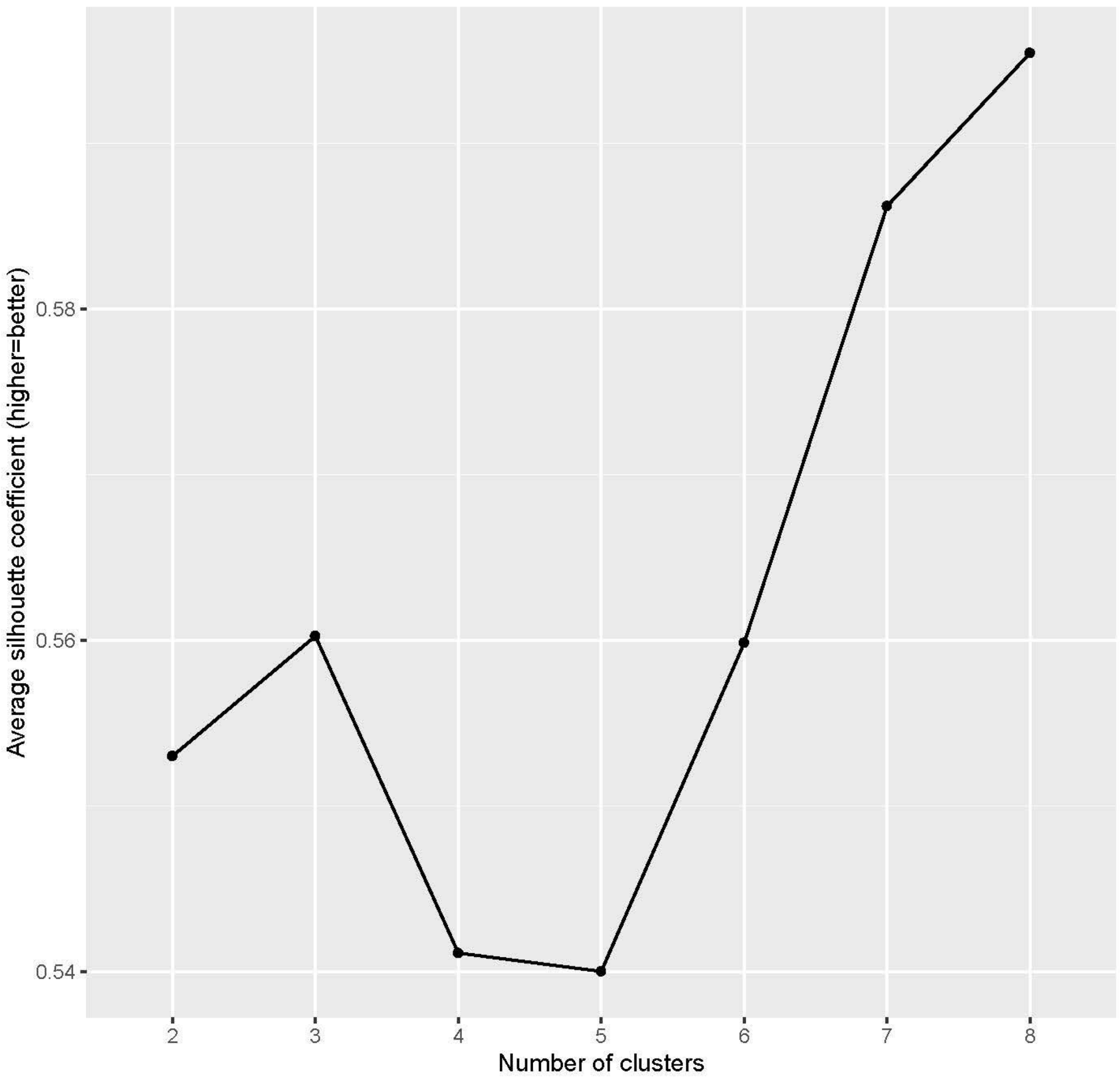
Average silhouette coefficient (higher=better)
0.58
0.56
0.54
2
3
4
5
6
7
8
Number of clusters
